# Cancer associated variant enrichment CAVE, a gene agnostic approach to identify low burden variants in chronic lymphocytic leukemia

**DOI:** 10.1038/s41598-024-73027-1

**Published:** 2024-09-20

**Authors:** Adar Yaacov, Gregory Lazarian, Tatjana Pandzic, Simone Weström, Panagiotis Baliakas, Samia Imache, Valérie Lefebvre, Florence Cymbalista, Fanny Baran-Marszak, Shai Rosenberg, Thierry Soussi

**Affiliations:** 1 Gaffin Center for Neuro-Oncology, Sharett Institute for Oncology, Hadassah Medical Center and Faculty of Medicine, Hebrew University of Jerusalem, Jerusalem, Israel; 2 The Wohl Institute for Translational Medicine, Hadassah Medical Center and Faculty of Medicine, Hebrew University of Jerusalem, Jerusalem, Israel; 3 Laboratoire d’hématologie, Hôpital Avicenne, Hôpitaux Universitaires Paris Seine- Saint-Denis, Bobigny, France; 4 INSERM, UMR 978, Université Sorbonne Paris Nord, Bobigny, France; 5 Department of Immunology, Genetics and Pathology , Uppsala University, Uppsala, Sweden; 6 Clinical Genomics Uppsala, Science for Life Laboratory, Uppsala University, Uppsala, Sweden; 7 Équipe Développement hématopoïétique et leucémique, Sorbonne Université, INSERM, Centre de Recherche Saint-Antoine, UMRS_938, CRSA, AP-HP, SIRIC CURAMUS, 27 rue de Chaligny, 10 éme étage, 75012 Paris, France; 8 Sorbonne Université, Place Jussieu, Paris, France

**Keywords:** Low-frequency genetic variants, TP53, Chronic lymphocytic leukemia, Computational tool, Cancer genetics, Haematological cancer, Tumour-suppressor proteins

## Abstract

Intratumoral heterogeneity is an important clinical challenge because low burden clones expressing specific genetic alterations drive therapeutic resistance mechanisms. We have developed CAVE (*cancer-associated variant enrichment*), a gene-agnostic computational tool to identify specific enrichment of low-burden cancer driver variants in next-generation sequencing (NGS) data. For this study, CAVE was applied to *TP53* in chronic lymphocytic leukemia (CLL) as a cancer model. Indeed, as TP53 mutations are part of treatment decision-making algorithms and low-burden variants are frequent, there is a need to distinguish true variants from background noise. Recommendations have been published for reliable calling of low-VAF variants of TP53 in CLL and the assessment of the background noise for each platform is essential for the quality of the testing. CAVE is able to detect specific enrichment of low-burden variants starting at variant allele frequencies (VAFs) as low as 0.3%. In silico *TP53* dependent and independent analyses confirmed the true driver nature of all these variants. Orthogonal validation using either ddPCR or NGS analyses of follow-up samples confirmed variant identification. CAVE can be easily deployed in any cancer-related NGS workflow to detect the enrichment of low-burden variants of clinical interest.

**Supplementary Information:**

The online version contains supplementary material available at 10.1038/s41598-024-73027-1.

## Introduction

Next generation sequencing (NGS) has rapidly expanded into the clinical setting in cancer research^1,2^, dramatically decreased the cost of large-scale sequencing by several orders of magnitude, and brought considerable advancements to diagnoses and treatment selection.

For patients with symptomatic chronic lymphocytic leukemia (CLL) harboring TP53 abnormalities, targeted therapies alone or in combination are more effective than immunochemotherapy and therefore represent the preferred option for these patients. These alternative approaches may include inhibitors of the B-cell receptor signaling pathway (ibrutinib, acalabrutinib, idelalisib and duvelisib) and of the anti-apoptotic protein BCL-2 (venetoclax)^3–6^.

A lower limit of detection (LOD) is an essential feature of using NGS in the clinic to detect low VAF variants that could affect the outcomes of the disease. LOD refers to the lowest level of genomic variants that a platform can detect reproducibly on a background of wild-type sequences. Although a variant allele frequency (VAF) as low as 2% can be used, this parameter is usually more so in the 5–10% range for most validated clinical NGS platforms, depending on the NGS workflow and the type of genomic change being detected^7^. Unfortunately, the use of NGS for the analysis of specific mutations lacks standardization for such aspects as the choice of variant callers, the minimum coverage depth, or the LODs used for variant calls^8,9^. That must change, as standardization is essential for low burden mutations with clinical impacts (e.g., *TP53* mutations in several hematological neoplasms (CLL or myelodysplastic syndromes, MDS)), the follow-up of minimal residual disease, or the detection of clonal hematopoiesis or circulating nucleic acids in sera or plasma.

Based on these data, current guidelines by the European Research Initiative on CLL (ERIC) warrant integration of *TP53* mutation analysis into the evaluation of CLL patients before treatment initiation. First published in 2012, these guidelines had been largely based on the use of conventional Sanger sequencing^10^. In 2018, ERIC published an update of their recommendations, taking into account the increasing use of NGS in clinical laboratories^11^. Nevertheless, the LOD for reporting *TP53* variants defined in these recommendations (VAF > 10%) was unsatisfactory, as it is now well established that driver *TP53* variants can be identified using lower LODs^12–18^. Importantly, the specific clinical impact of these *TP53* mutated minor clones remains unclear, with the results of studies pertinent to this question remaining controversial, possibly due to the various tools used for *TP53* variant detection and validation or the heterogeneity of the cohorts used in these studies. This issue has been partially solved in the recent release of the 2024 recommendations update^19^. No LOD cut-off for reporting TP53 mutations were recommended. Instead, reporting laboratories will need to define and validate their own procedures. These procedures are highly heterogenous including either serial dilution of TP53 variants that address only a few positions among the large distribution of p53 variant that covers most entirely the TP53 gene, repeated sequencing or orthogonal validation that increases the costs of the analysis.

In the present study, we developed an innovative and versatile computational analysis based on the use of raw sequencing data and data on several thousand oncogenic *TP53* variants in a range of data repositories to differentiate sequencing errors from true pathogenic variants.

## Results

### Low-VAF pathogenic TP53 variants are common in CLL and found preferentially in tumors with high intratumoral heterogeneity

First, a pooled analysis was performed on the six publications that have addressed the clinical value of low-burden clones. This analysis employed novel classification tools specifically developed for *TP53* and based on the UMD database (Materials and methods and Supplementary Table S2)^12–15,17,20^. Gathering 1,007 *TP53* variants with well-defined VAFs from these patients cohorts (untreated or treated) and analyzing them with different methodologies led to specific observations that were not apparent in individual studies (Supplementary Fig. S1 to S5). Using *TP53*-specific ACMG criteria included in UMD_*TP53*, the great majority of these variants could be classified as pathogenic (P) or likely pathogenic (LP). Also, more than 50% of them were indeed certified oncogenic variants, based on the CSD *TP53*-specific classification (ranges 55 to 76 depending on the study) (Materials and methods and Supplementary Fig. S1A and B). Pooling VAF data from these studies showed that pathogenicity predictions for low-VAF variants (less than 5%) were similar to those for high VAF variants. Furthermore, distribution was similar for variants included in the CSD dataset or ACMG classified as P, LP or as variants of uncertain significance (Supplementary Figs. S2A and B and S3A and B). Considered together, these findings suggest that no methodological artifacts were selected by lowering the LOD and that true variants, whatever their classification, can be found at high or low VAF. This analysis also confirmed the important intratumoral heterogeneity of *TP53* mutations, with 53% of patients carrying more than one *TP53* variant (Supplementary Fig. S4A and B) and an inverse correlation between the VAF and the number of *TP53* variants per patient (Supplementary Fig. S5A). This pooled analysis also showed that low-burden clones are predominantly found in patients with multiple *TP53* variants (Supplementary Fig. S5B). Multiple strategies were used to define the LOD used in these studies, including calibration with specific *TP53* variants, repeated sequencing and various heterogenous statistical pipelines, many of which are costly or not suitable for routine clinical platforms. Then, using both standard NGS data resulting from routine analyses of CLL patients recruited in the Avicenne Hospital (France, 2015–2021) and data referenced in open-access databases of annotated cancer variants, we developed a reproducible and portable computational analysis to differentiate sequencing errors from true pathogenic variants. Data used in this study were acquired using an Ion Torrent platform widely used for clinical testing. This method can, however, be easily adapted to any other sequencing methodologies.

### Cancer-associated TP53 variants are abundant in low-VAF calls

**Fig. 1:**
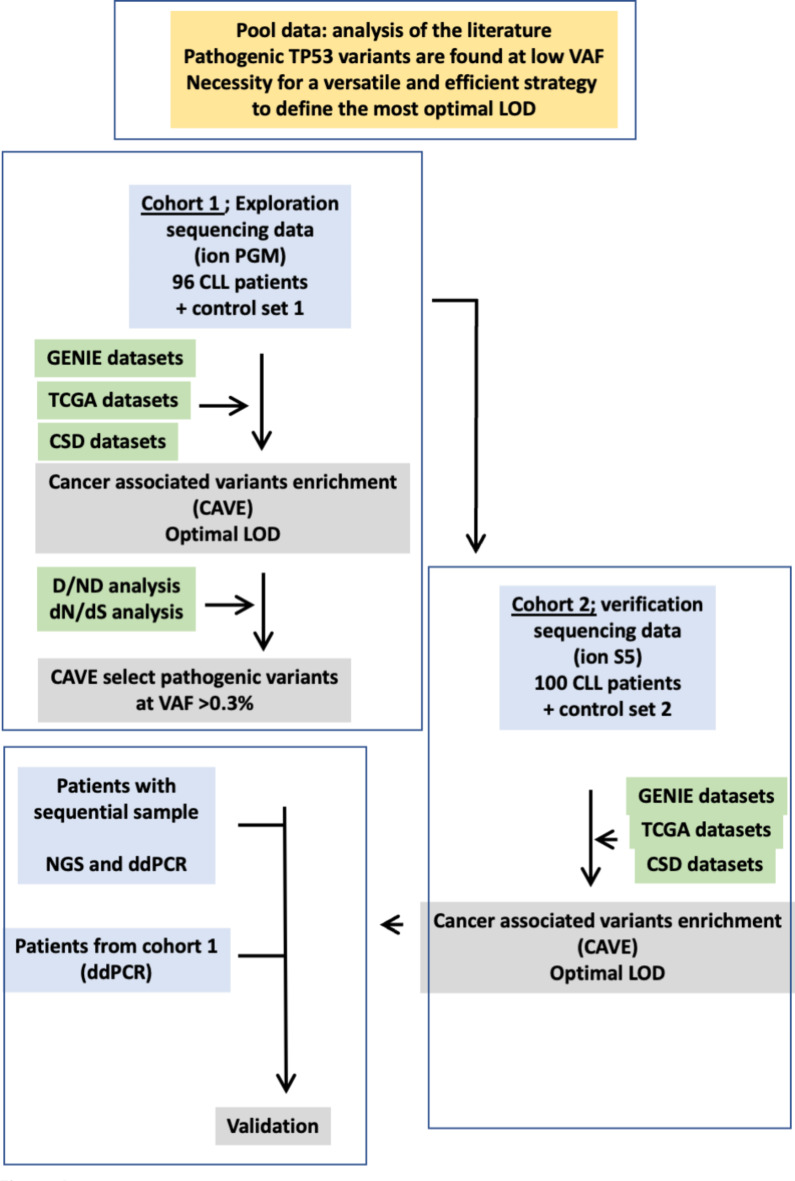
Flow chart of the strategy used to define the most optimal limit of detection. Two datasets from 96 (exploratory cohort) and 100 (verification cohort) CLL patients were analyzed.

Two sets of *TP53* NGS data obtained from routine analyses of 196 CLL patients were used (Materials and methods and Fig. 1). Although the recommended cut off to report TP53 mutation in CLL was previously defined to 10% and would have led to the clinical management of 32 patients, lowering this value to either 5 or 1% allowed the detection of 4 and 16 TP53 mutated patients respectively confirming the presence of a significant number of patients with only low burden variants (Supplementary Fig. S6A). These low burden variants include hot spot mutations and are predominately classified pathogenic or likely pathogenic indicating that lowering the LOD does not lead to the selection of spurious variants (Supplementary Fig. S6B–D).

Analysis of the distribution of low-VAF calls (VAF range 0.05–5%) from the first set (96 patients, exploration cohort) showed a specific enrichment of variants with VAFs higher than the background noise in exons 4 to 9, the *TP53* region commonly mutated in various types of cancer (Supplementary Fig. S7A). No enrichment was observed in exon 9 beta or gamma expressed by the two *TP53* isoforms untargeted by cancer-associated mutations, or in the unfrequently mutated exons 2, 3 and 11 (Supplementary Fig. S7). This particular distribution was not observed in the control data obtained from the repeated sequencing of DNA from the CLL cell line HG3 (unmutated *TP53*) (Supplementary Fig. S8A). Because this specific distribution was not dependent on exon or amplicon size (Supplementary Fig. S9A–C), it is likely that low-VAF pathogenic variants were included in this dataset gathered from CLL patients. It is usually assumed that early and random PCR errors are the principal source of NGS noise for SNVs. However, in this study, the mutational landscape of extremely low VAFs (below 0.5%) was similar to the spectrum of random errors generated by the Taq polymerase with a predominance of AT > GC substitutions (Supplementary Fig. S10A–F). The landscape of VAFs between 0.5 and 1% was intermediary, suggesting the selection of specific variants. For variants found at frequencies higher than 1%, the profile was similar to the 4700 *TP53* pathogenic variants found in CLL patients (Supplementary Fig. S9A and D). Altogether, this analysis confirmed that a significant number of non-random variants are found in a VAF range of 0.5–5% and enriched in *TP53* regions commonly mutated in tumors.

**Fig. 2:**
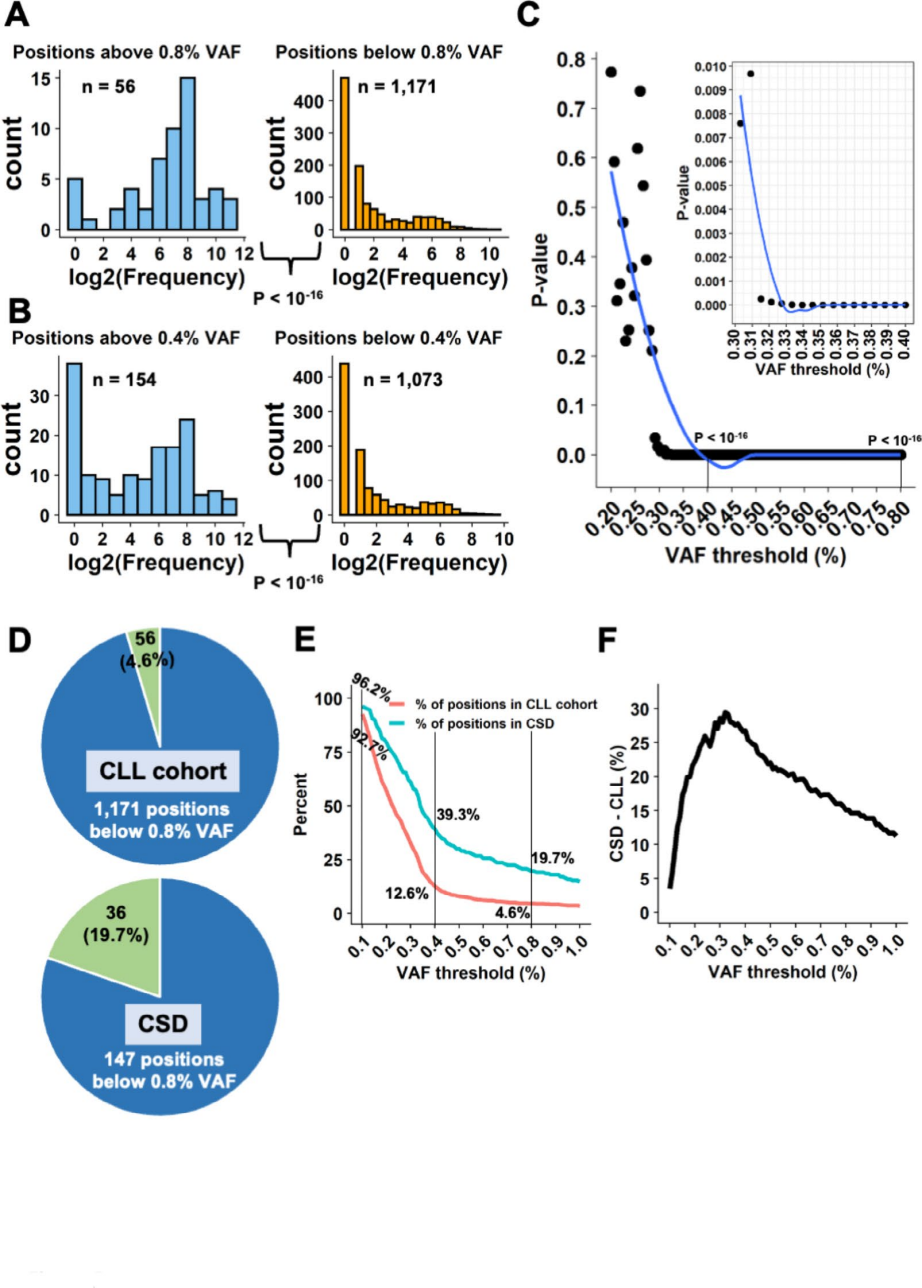
Cancer-associated *TP53* variants are abundant in low-VAF calls. (**A**) Histograms showing frequency of genomic positions in GENIE (i.e., number of times mutations at the specific position has been reported in the database), illustrating positions above (left) and below (right) a 0.8% VAF threshold. X-axis, log2(frequency + 1). P-value derived from a two-sided Wilcoxon rank-sum test. (**B**) Similar frequency histograms for a 0.4% VAF threshold. (**C**) Generalization of the test for multiple, numerous, VAF thresholds between 0.2% and 0.8%. Each point represents a test as presented in A and B. Y-axis, p-value, two-sided Wilcoxon rank-sum test. (**D**) Pie charts showing enrichment of low-VAF calls in CSD. Above 0.8% VAF, 4.6% (56/1,227) of the CLL cohort positions include 19.7% (36/183) of CSD positions. (**E**) The red curve represents the percent of positions found above the relevant threshold and the turquoise curve how many of them are found in CSD relative to the CSD size of 183 positions. (**F**) Difference of percentages from **E**. A higher difference means that more CSD positions were explained by the above threshold positions found in the cohort, controlling for the number of positions.

We devised a strategy to identify the VAF best able to separate true cancer-associated variants from background noise. *TP53* is the most frequently mutated gene in human cancer and there are thus numerous, highly curated and independent repositories of *TP53* cancer-associated variants available via large-scale tumor genome sequencing projects such as GENIE, TCGA, or ICGC (see Materials and methods for details). Because the number of novel *TP53* missense variants has not increased significantly for several years now, it is assumed that a saturation plateau has been reached, with the discovery of all potential *TP53* variants that sustain a defect in the protein’s tumor suppressor function^21^. It is therefore possible to use database frequency as a proxy to identify potential driver variants. The frequency of each position in the sequencing data collected from the exploration cohort was retrieved from the GENIE database, defining *cancer-associated variant enrichment* (CAVE), a proxy for potential driver—and therefore pathogenic—variants. Indeed, a high CAVE value should be associated with hotspot positions and a low or null value with infrequent or absent variants. Therefore, it was possible to analyze CAVE distribution according to the VAF of each position, as shown in Fig. 2A. Splitting sequencing data into two groups according to a specific VAF generated two CAVE distributions that were used to identify the optimal VAF cut-off for cancer-associated variants (Fig. 2A and B). Above a VAF of 0.8%, the CAVE distribution showed strong enrichment for cancer-associated *TP53* variants, whereas below it, the CAVE distribution was largely squeezed toward rare or non-mutated *TP53* positions. The difference between the two distributions was highly significant (*p* < 10^−16^, two-sided Wilcoxon rank-sum test). A similar analysis using a lower VAF value (0.4%) provided similar results (*p* < 10^−16^, two-sided Wilcoxon rank-sum test) (Fig. 2B). This analysis was therefore repeated for all VAFs ranging from 0.2 to 0.8% (Fig. 2C). P-values for the different VAF thresholds showed a qualitative behavior of an exponential decay curve, and a curve “knee” was noted between the VAF thresholds of 0.3% and 0.35% (Fig. 2C). The p-values were not significant for VAF values below 0.3% but they decreased sharply above that threshold (Fig. 2C). To confirm that finding, another repository of *TP53* variants was used, specifically one from the TCGA comprising data not overlapping with the GENIE datasets. A similar CAVE distribution with the same curve knee around 0.3% was observed in that confirmatory analysis (Supplementary Fig. S11A).

Finally, the CSD was used, i.e., a highly validated set of cancer-associated mutations in *TP53*(Materials and methods)^22^. Out of 1,227 different genomic positions in the CLL cohort, 183 positions were found in the CSD. For the example threshold of 0.8% VAF, which consists of 56 different positions in group 1 (above threshold), 36 positions were mutual to the CSD, meaning that 4.6% of the cohort positions (56/1,227) included 19.7% of the CSD positions (36/183) (Fig. 2D). Hence, the > 0.8% VAF threshold group was enriched with positions of a set of highly validated pathogenic mutations in *TP53*, with a difference of 19.7–4.6% = 15.1%. The enrichment of positions from the CSD was then tested at a variety of different VAF thresholds. The largest CSD abundance compared to the group’s size was found around a 0.30–0.35% VAF threshold, similar to the informative threshold found in the analysis described above with reference to the GENIE database (Fig. 2E-F).

A second group of 100 CLL patients from the same clinical center was used as a verification cohort to evaluate the performance of the new Ion Torrent sequencer (Materials and methods). Strikingly, the analysis of the verification cohort showed the same profile, further strengthening 0.3–0.4% as an informative threshold (Supplementary Fig. S7, S11B and C). In contrast, the analysis of a second control dataset obtained from cancer-free individuals did not generate a specific profile (Supplementary Fig. S8C,D).

Considered together, these results obtained via two different cohorts of patients sequenced using different devices, but the same methodology showed reproducibly and without bias that there was a strong enrichment of cancer-associated variants at VAFs above 0.3%.

### Independent confirmation of the pathogenicity of low-VAF cancer-associated variants

**Fig. 3:**
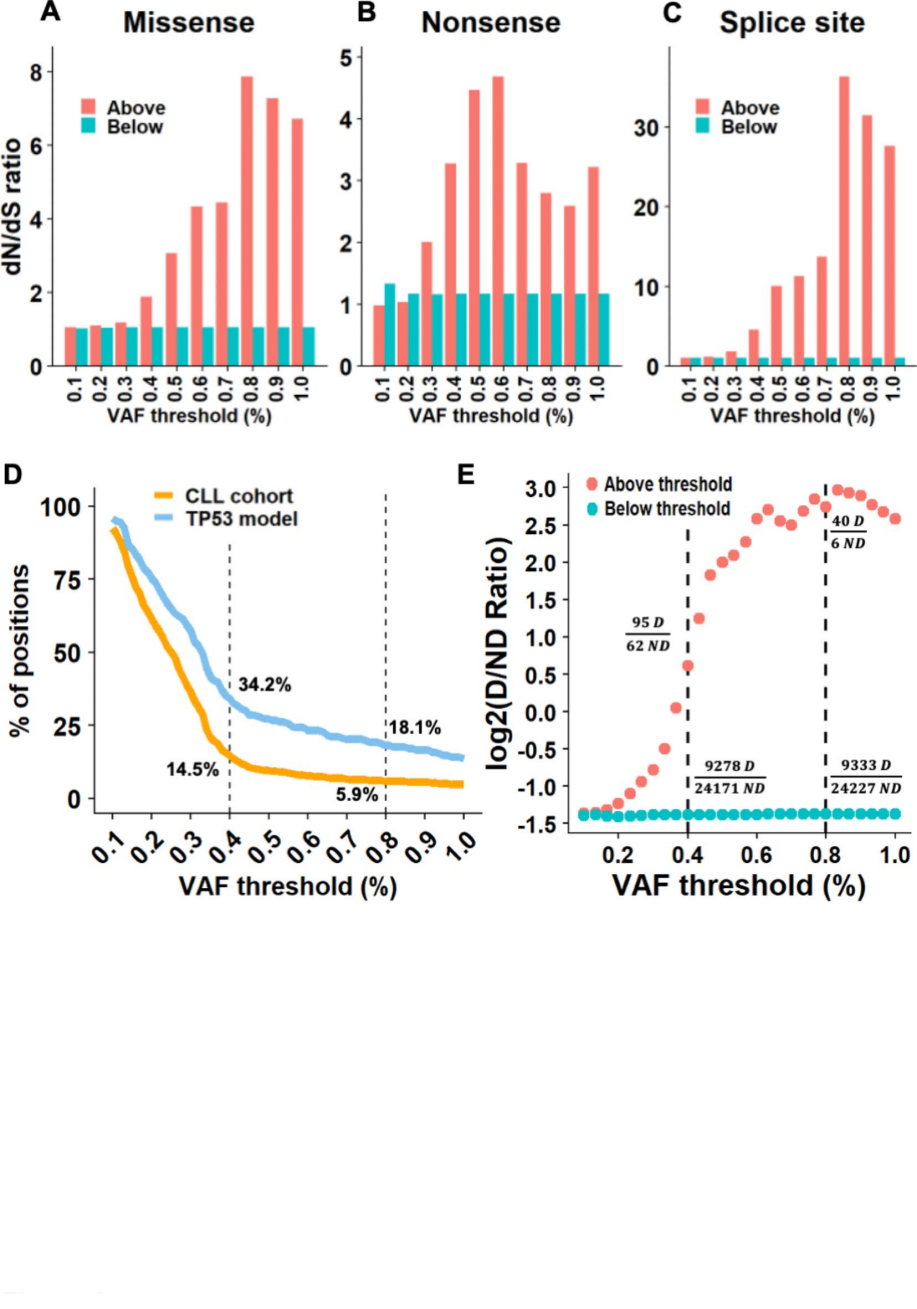
CAVE analysis reveals variants under positive selection in CLL. (**A–C**) Bar plots showing dN/dS results for missense (left), nonsense (middle) and splice site (right) mutational events (data from the exploration cohort). (**D**) Curves similar to those in Fig. 2E, for positions in *TP53* missense mutation categorized as deleterious by *TP53*_PROF. (**E**) Ratio of deleterious/non-deleterious missense mutations per *TP53*_PROF, at various VAF thresholds. For each point, the ratio was calculated once for the above calls (red) and once for the calls below (turquoise). Y-axis, Log2(ratio + 1). D: deleterious; ND: non-deleterious.

One of the most common ways to measure positive selection of mutations is the non-synonymous to synonymous ratio (dN/dS). We used dNdScv^23^ to test this study’s calls for positive selection at different VAF thresholds (Materials and methods). The dN/dS ratio was calculated at each VAF threshold for missense, nonsense, and splice-site mutations, once for calls above and once for calls below the concerned threshold. At all VAF thresholds > 0.3%, the dN/dS ratio was higher than 1 in the above-threshold calls and ~ 1 in the below-threshold calls (Fig. 3A). Interestingly, this observation held true for both nonsense and splice-site mutations as well (Fig. 3B,C). The calls both above and below VAF thresholds of 0.1–0.2% had ratios of ~ 1, meaning they presented no positive selection.

We recently developed *TP53*_PROF, a *TP53* machine-learning model able to predict the pathogenicity of any possible missense *TP53* variants with 96.5% accuracy^24^. First, similarly to the CSD analysis presented above, we assessed enrichment of genomic positions, in which at least one deleterious mutation was predicted by the model, above different VAF thresholds. Once again, the largest enrichment was found at a VAF of ~ 0.4% (Fig. 3D). Next, the calls were annotated according to the model predictions. Of the total of 56,219 calls, 33,606 were missense mutations. For each threshold between 0.2 and 0.8% VAF, the deleterious/non-deleterious (D/ND) ratio was calculated, for calls both above and below the VAF threshold (Fig. 3E). For those above any VAF threshold ≥ 0.4%, there were more deleterious than non-deleterious mutations. For example, at VAF thresholds of 0.4% and 0.8%, the D/ND ratios were 1.53 and 6.67 respectively (Fig. 3E). All of these observations were confirmed by the analysis of the validation cohort (Supplementary Fig. S12A to E).

Only zero to five non-deleterious mutations were found at VAF thresholds ≥ 1% and none were found at VAF thresholds above 2%. The D/ND ratios below different VAF thresholds were consistently near 0.384 (0.377–0.386), meaning that there were > 2.5 times more benign mutations than pathogenic ones below any VAF threshold (Fig. 3E). These results suggest that most cancer-associated variants identified above a LOD of 0.4% are indeed pathogenic.

### Orthogonal validation of low-call variants via ddPCR

**Fig. 4:**
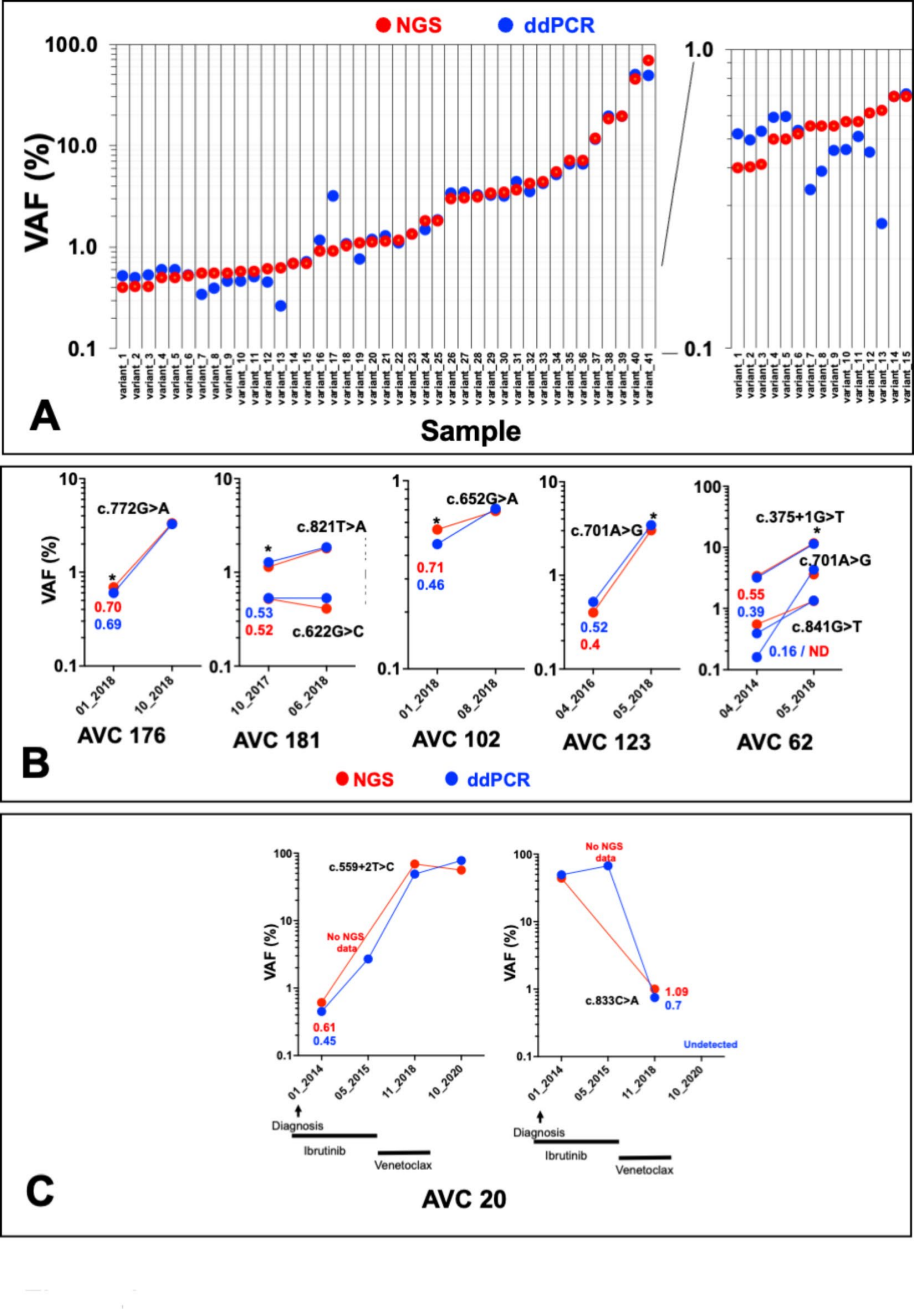
Orthogonal validation of low-VAF variant via ddPCR and analysis of sequential sample. (**A**) Samples from the exploration cohort were analyzed via ddPCR. The right panel shows variants with VAFs lower than 1% using a different scale. (**B**) Follow-up of patients from the verification cohort (labeled with a star). Samples were analyzed both by NGS and ddPCR. (**C**) Patient AVC20.

Orthogonal ddPCR was carried out to validate low-VAF variants. To prevent any bias associated either with position in *TP53* or with specific mutational events, 21 different *TP53* variants identified in 16 samples were analyzed (Fig. 4A). Except for two variants found at low frequency via NGS (VAF 0.15%), total concordance was observed between ddPCR and NGS including samples with NGS VAFs between 0.4% and 1% (R^2^ = 0.9332; *p* < 0.0001) (Fig. 4A and Supplementary Fig. S13). For five patients from the exploration cohort, sequential samples collected at different times during disease progression were available and analyzed via NGS and ddPCR. For three of them (AVC 176, 181 and 102) low-VAF variants (below 1%) were confirmed in follow-up samples (Fig. 4B). For two (AVC 123 and AVC 62), the same variants were found in samples taken several years before the one used in the exploration cohort (Fig. 4B). NGS data were validated by ddPCR for all but one (ddPCR VAF 0.16%) of these variants. In 2018, patient AVC 20, not included in the exploration cohort, was found to have two *TP53* variants (one splice variant, c.559 + 2, NGS VAF 49% and one missense variant, c.833 C > A, NGS VAF 1%) (Fig. 4C). NGS and ddPCR analysis of four sequential samples for this patient showed that the missense variant found at low frequency in 2018 was predominant at the time of diagnosis in 2014. The splice variant was also detected in 2014 but at low frequency (NGS VAF 0.61%). Treatment with the BTK inhibitor ibrutinib brought about the elimination of the clone expressing the missense variant, but the minor clones expressing the splice variant become predominant and remained present despite treatment with the BCL2 inhibitor venetoclax (Fig. 4C).

Taken together, the ddPCR validation and the investigation on sequential samples confirmed the CAVE analysis and the identification of low-VAF clones.

## Discussion

In cancer, somatic mutations lead to the production of heterogeneous tumors with multiple high and low burden mutations. Because of its high-throughput and massively parallel sequencing capabilities, NGS has become the preferred methodology for routine testing in clinical molecular diagnostic laboratories. Defining an optimal LOD in clinical NGS platforms is becoming essential for the detection of low-level mutations^1,25^. This has pertinence, for example, in samples with limited tumor content or clonal heterogeneity, for the monitoring of therapy responses (defining minimal residual disease) and the screening of circulating nucleic acids. Unfortunately, most NGS pipelines suffer from subpar performance for the detection of low-VAF variants (less than 5%) due to sequencing artifacts originating from sample processing, the sequencing methodology or data analysis.

Deletion of 17p involving the loss of TP53 gene and/or the mutations in TP53 are identified in 4–10% of patients at diagnosis^3^ but can be acquired throughout the disease course, with an estimated prevalence of 40% in refractory CLL^26^. Even in cases when this abnormality only occurs in a small fraction of the neoplastic cells, the presence of subclonal TP53 alterations have been shown to carry poor prognosis^27^. Currently, *TP53* alteration in CLL is an essential marker for the initiation of novel therapeutic options, such as ibrutinib/idelalisib/acalabrutinib or venetoclax, targeting B-cell receptor signaling or BCL-2, respectively. These findings highlight the importance of identifying patients with TP53 abnormalities and led to the recommendation to include evaluation for TP53 abnormalities into routine workup for CLL^10,11,19,28^. The new recommendations defined by ERIC require each laboratory to define its own procedure for validating the optimal cuf-of LOD associated with the sequencing method.

A number of workflows have been deployed across a range of publications in the setting of CLL to identify low-VAF *TP53* variants but the clinical value of these latter remains controversial (review by^27^). Although, most of these variants are pathogenic (Supplementary Figs. S1 to S5), the clinical use of low-VAF variants is still pending for several reasons. First, methods and pipelines used to detect and validate these low-VAF variants are still very heterogenous and not suitable for routine practice. Second, how low-VAF variants will behave during disease progression remains unclear, an aspect that may be partly associated with methodological biases.

In the present work, using raw data obtained from routine clinical analyses, we developed a robust pipeline to assess cancer-associated variant enrichment with the goal of defining the most optimal LOD. This methodology, named CAVE, was built upon the use of independent open-access repositories of hundreds of thousands of oncogenic *TP53* variants identified in multiple cancers^6,29^. With the CAVE methodology, we were able to identify pathogenic variants above nonspecific background noise. We validated the low-burden *TP53* variants identified through CAVE using either *TP53*-dependent (*TP53* PROF or CSD) or *TP53*-independent (dN/dS) methods. Finally, both ddPCR and the sequencing of longitudinal samples validated the CAVE results, showing not only that low-VAF *TP53* variants identified by CAVE are observed in tumors, but also that their burden increases over the years during tumor progression. This important dynamic of *TP53* variants in CLL warrants the accurate identification of these low-VAF clones to empower investigations and tailor the best therapeutical regimen for patients with the disease. Considering our recent analysis of *TP53* mutations in CLL patients and literature data, we estimate that at least 20% of *TP53* variants have a VAF lower than 5% and thus patients with them may not receive pertinent therapy.

CAVE includes several features that make it easy to use in clinical laboratories. It is based on the use of readily available sequencing data produced during clinical analyses (e.g., MAF or VCF files) with no need for costly sample resequencing. Furthermore, it is totally platform agnostic and therefore allows users to define their own LODs based on the background of the sequencing method. Although the present study focuses on *TP53* variants in CLL, CAVE can be extended to AML or MDS where *TP53* mutations are used not only as a prognostic marker but also as a target to define minimal residual disease.

CAVE is designed to be portable and therefore applicable to any gene as long as variant repositories exist for it. This is the case for most cancer genes: currently, more than 200,000 cancer genomes are available, and this number will grow steadily in the coming years. That growth will, furthermore, increase the recurrence rate of cancer-associated variants, which is one of the strongest and most unbiased criteria to prioritize pathogenic variants used by CAVE. Furthermore, the use of the gene-agnostic non-synonymous to synonymous substitutions (dN/dS) ratio for variant validation makes CAVE highly polyvalent.

NGS technology is widely used in clinical laboratories for the analysis of tumor materials, but its utilization in infectious disease had remained quite rare until the recent COVID-19 pandemic, which energized the sequencing of hundreds of thousands of SARS-CoV-2 samples^30^. The high mutability of the virus makes each sample of it similar to a tumor sample, both showing important intra-sample heterogeneity. Furthermore, the availability of several billion SARS-CoV-2 genome sequences will provide a strong comparison reference that can be used for CAVE analyses.

In summary, we developed a methodology to process NGS sequencing data. Called CAVE, the methodology can be easily applied to any custom or commercial gene panel. CAVE can be directly applied to sequencing data to evaluate background errors and identify variants of clinical significance.

## Materials and methods

### Sequencing data

TP53 data were extracted from VCF files derived from CLL patients analyzed in routine care in Hôpital Avicenne (Online Supplemental Methods). Sequencing was performed using two different systems, the Torrent PGM semiconductor system (Ion PGM Hi-Q Sequencing Kit, Thermo Fisher) for the analysis cohort (96 patients) and the Ion S5 XL system (Ion 510 & Ion 520 & Ion 530 Kit – Chef, Thermo Fisher) the verification cohort (100 patients). Although, the minimum allele frequency thresholds applied in the variant callers parameter settings was 1% in the routine setting, for the development of CAVE, the variant callers parameter settings was 0.05% allowing the detection of an average of 1000 variants called per sample.

### Databases

*TP53* mutations from various independent repositories (TCGA, GENIE or UMD) were downloaded from their respective websites (Supplementary Table S1).

### CAVE analysis

Since the first publication of TP53 mutations in 1989, more than 250 000 TP53 mutated tumors have been described and collected in various databases such as UMD_TP53, GENIE, TCGA or IARC^6,31,32^. Chronological analysis of TP53 variants published in the course of 33 years shows that no new missense variants are now described suggesting that a saturation plateau has been reached with the identification of all potential defective TP53 variants. This issue is supported by the finding that TP53 missense variants that have never been observed in human cancer have been shown to retain wild type TP53 function^29,33^.

CAVE was optimized to handle VCF files issued from NGS platforms. In all subsequent analysis, only SNV variants have been taken into account. For each position in TP53, the frequency of single nucleotide variation in the cohort was compared to the same information issues from the GENIE database that include only cancer associated variants. This measure indicates how many times a mutation in each genomic position has been observed in a tumor. Then, VAF cut-off thresholds can be examined. For each VAF threshold selected, two groups were defined: (i) The upper group including variant positions for which VAF was above the threshold in at least one patient, and (ii) the lower group including the remaining position identified in the cohort. Thereafter, each position was assigned with a frequency score (defined by prevalence in the GENIE database). Two-sided Wilcoxon rank-sum tests were performed to compare the frequency differences between the two groups. Statistically significant differences in the frequency distributions between the both groups imply that one group (the upper one) is enriched with cancer-associated variants relative to the other. The p-value and the trends in the p-value change across VAF thresholds (i.e., dynamics of different VAF thresholds) are then used to detect optimal cut-offs. Validation of this strategy has been performed by using TCGA data, an independent cancer mutation database.

### Variants validation

Non-synonymous to synonymous ratio (dN/dS) analysis was carried out using dNdScv, an R package with a group of maximum-likelihood dN/dS methods designed to quantify selection in cancer as described in supplementary methods^23^. Validation using the Cancer Shared Datasets (CSD) of TP53 variants is described in the online supplementary Methods. TP53*_PROF is* a machine-learning model that classifies all possible *TP53* missense mutations as either deleterious or non-deleterious with 96.5% accuracy^24^. Each missense mutation in the data was assigned a label of D or ND for deleterious or non-deleterious, respectively. Different VAF thresholds were explored both in a group-wise manner of above and below VAF thresholds, and in a patient-specific manner.

### Statistical analyses

Statistical analyses were performed using R version 4.1.0. Two-sided Wilcoxon rank-sum tests were performed with the “Wilcox.test” function in R. All boxplots are presented according to the standard boxplot notation in R (ggplot2 package): the center line marks the median value; top and bottom limits mark first and third quartiles; and whiskers cover data within 1.5x the interquartile range from the box.

## Electronic supplementary material

Below is the link to the electronic supplementary material.

Supplementary Material 1

Supplementary Material 2

Supplementary Material 3

## Data Availability

Data supporting the study are available in the manuscript and its supplementary information. TP53 NGS data of exploration and verification cohorts are available from the corresponding author upon reasonable request. Details about the 6 publications used for a pooled analysis are in Table S1, and publicly available cancer NGS datasets used in this study are listed in detail in Table S2. Cave scripts and sequencing data used for the analysis are avaialble from GitHub: https://github.com/Adarya/CAVE.
