# Supplementary material for: Cancer associated variant enrichment CAVE, a gene agnostic approach to identify low burden variants in chronic lymphocytic leukemia
Source: Sci Rep. 2024 Sep 20;14:21962. doi: 10.1038/s41598-024-73027-1 (PMC11415367; doi:10.1038/s41598-024-73027-1)
Supplement: Supplementary file 1 — Supplementary Material 1 [file 41598_2024_73027_MOESM1_ESM.docx]

**Supplementary Methods**

*Patient and TP53 mutation analysis by deep NGS*

PBMC were isolated from CLL samples with monoclonal B cells blood count greater than 10G/L identified by flow cytometry. The PBMC used in this study were generally greater than 90% CLL B cells to avoid VAF underestimation as minor clones are part of the tumoral population. DNA was extracted from peripheral blood mononuclear cells with a QIAcube instrument (Qiagen, Les Ulis, France). The Ion AmpliSeq *TP53* community panel (Thermo Fisher, Illkirch France) designed to analyze all coding exons of the *TP53* gene including alternative exons beta and gamma was used for all patients. Libraries were prepared from 10 ng of DNA with the Ion Chef System (Thermo Fisher). Sequencing according to the manufacturer’s protocols on the Ion Torrent PGM semiconductor system (Ion PGM Hi-Q Sequencing Kit, Thermo Fisher) and on the Ion S5 XL system (Ion 510 & Ion 520 & Ion 530 Kit – Chef, Thermo Fisher) for respectively the exploration and verification cohorts enabled the attainment of an average depth of 10 000X for both. BAM alignment and variant calling were done using Torrent Suite Software version 5.6 for the PGM and 5.12.0.2 for the S5 XL platforms. The minimum allele frequency thresholds applied in the variant callers parameter settings was 1% in the routine setting (1).

*Droplet digital PCR*

The QX200 AutoDG system (Bio-Rad) was used for droplet digital PCR (ddPCR) assay analysis. Primers/Probes for the different *TP53* variants were designed using the Bio-Rad online tool for ddPCR. Consumables and reagents for ddPCR were purchased from Bio-Rad and used according to the manufacturer’s instructions. In short, the PCR reaction mix was prepared in 22 µL using 1x ddPCR Supermix for Probes (no dUTP), 900 nM primers, 250 nM for each probe (FAM and HEX) and 40 ng DNA. The reaction was then partitioned into 20,000 droplets using the AutoDG, followed by PCR with a 55°C annealing/extension temperature. The droplets were then read with the QX200 Droplet Reader (Bio-Rad). Analysis of ddPCR data was done with QuantaSoft Analysis Pro software (Bio-Rad).

*Cancer Shared Dataset analysis*

We recently developed a novel concept to define *TP53* oncogenic driver variants based on their simultaneous occurrence in independent large-scale datasets such as UMD_*TP53* (variants defined by Sanger sequencing only), TCGA, ICGC and MSKCC Impact (2). Analysis of all *TP53* variants included in this Cancer Shared Dataset (CSD) using the various large-scale functional assays available confirmed that all these variants have lost their tumor suppressive function. Although the high stringency of this selective procedure does not enable the capture of all oncogenic *TP53* variants, it does lead to the selection of *TP53* variants that can be defined non-ambiguously as not functional (2). *TP53* variants defined using this procedure have been successfully deployed as a positive training set to develop *TP53* PROF (prediction of functionality), a gene-specific machine-learning model to predict the functional consequences of every possible missense mutation in *TP53* (3). Overall, 183 different genomic positions were found both in the CLL exploration cohort and in the CSD. An abundance test of calls in CSD was based on the relative share of CSD positions in a group of calls above a given VAF threshold. For example, in the CLL exploration cohort, there were 56 different genomic positions above 0.8% VAF, which represented 4.6% of the total cohort’s positions (56/1,227). Of these 56 positions, 36 were also found in the CSD. This latter has 183 different positions, hence, 4.6% of the CLL exploration-cohort positions included 19.7% (36/183) of CSD positions in the cohort – thus representing enrichment of deleterious variants above the threshold of 0.8%. This analysis was repeated for different VAF thresholds.

*Variants validation*

For Non-synonymous to synonymous ratio (dN/dS) analysis, the dNdSloc implementation was used (4). It infers the local mutation rate without exploiting additional information from other genes. This tactic was chosen because only *TP53* calls were used in the present work. dN/dS was calculated separately for missense, nonsense and splice site mutations, both for the above and below VAF threshold calls. Briefly, a ratio greater than 1 indicated positive selection, a ratio less than 1 negative selection, and a ratio equaling 1 no selection.

*Statistical analyses*

Statistical analyses were performed using R version 4.1.0. Two-sided Wilcoxon rank-sum tests were performed with the “Wilcox.test” function in R. All boxplots are presented according to the standard boxplot notation in R (ggplot2 package): the center line marks the median value; top and bottom limits mark first and third quartiles; and whiskers cover data within 1.5x the interquartile range from the box.

**References**

1. Sujobert P, Le Bris Y, de Leval L et al. The Need for a Consensus Next-generation Sequencing Panel for Mature Lymphoid Malignancies. Hemasphere 2019; 3:e169.

2. Soussi T, Leroy B, Devir M, Rosenberg S. High prevalence of cancer-associated TP53 variants in the gnomAD database: A word of caution concerning the use of variant filtering. Hum Mutat 2019; 40:516–24.

3. Ben-Cohen G, Doffe F, Devir M, Leroy B, Soussi T, Rosenberg S. TP53_PROF: a machine learning model to predict impact of missense mutations in TP53. Brief Bioinform 2022; 23:bbab524.

4. Martincorena I, Raine KM, Gerstung M et al. Universal Patterns of Selection in Cancer and Somatic Tissues. Cell 2017; 171:1029–1041.e21.
